# Material Properties and Morphology of Prestomal Teeth in Relation to the Feeding Habits of Diptera (Brachycera)

**DOI:** 10.3390/insects13020207

**Published:** 2022-02-17

**Authors:** Matthew S. Lehnert, Lauren A. Tarver, Jiansheng Feng

**Affiliations:** 1 Department of Biological Sciences, Kent State University at Stark, North Canton, OH 44720, USA; ltarver2@kent.edu; 2 School of Polymer Science and Polymer Engineering, The University of Akron, Akron, OH 44325, USA; jfeng@uakron.edu

**Keywords:** fly mouthparts, feeding habits, sclerotization, inorganic elements, elastic modulus, hardness

## Abstract

**Simple Summary:**

The prestomal teeth are structures on the mouthparts of some fly species that rasp surfaces to expose liquids for ingestion. Here, we investigated the material properties of prestomal teeth, including their hardness, elastic modulus, the extent of sclerotization, and elemental composition, and combined these results with morphology to determine their relationship to fly feeding habits. The results indicated that the prestomal teeth are heavily sclerotized and have hardness and elastic modulus values similar to the polymer polycarbonate. Although the presence of inorganic elements contributes to a harder cuticle in other insect species, the fly species studied here had only low concentrations of inorganic elements. We found evidence that the material properties and morphology of prestomal teeth relate to feeding habits, not phylogeny. In particular, flies that pierce tissues for liquids have larger prestomal teeth relative to their mouthpart sizes when compared to species that generally feed on exposed liquids. Flies are one of the most successful groups of organisms and their success likely relates to their ability to feed on a large array of nutritional liquids. Given their importance in ecology systems and relevance to medical and veterinary entomology, the functional morphology of fly mouthparts warrants additional studies.

**Abstract:**

Prestomal teeth are cuticular projections on the mouthparts of some fly species that rasp surfaces when feeding. Although prestomal teeth morphology has been reported for several fly species, their material properties have not been investigated. Here we report the morphology, elemental composition, extent of sclerotization, hardness, and elastic modulus of prestomal teeth and relate these findings to feeding habits. Scanning electron microscopy revealed that species categorized as flower visitors have a large labellum with numerous pseudotracheae and lack prestomal teeth, generalist species have these same features but with prestomal teeth, and specialist species that feed on blood or other insects have a smaller labellum with few or no pseudotracheae and relatively large prestomal teeth. Confocal microscopy revealed that prestomal teeth are heavily sclerotized and the labellum contains resilin, an elastomeric protein. Hardness and elastic modulus were explored with nanoindentation and showed that the insectivorous *Scathophaga stercoraria* had the hardest prestomal teeth and the highest modulus. Energy dispersive x-ray spectroscopy revealed that prestomal teeth had low concentrations of inorganic elements, suggesting that hardness might be partially supplemented by inorganic elements. Our findings indicate that prestomal teeth morphology and material properties relate more to feeding habits than to phylogeny.

## 1. Introduction

Insect mouthparts provide notable examples of structure-function relationships [1,2,3], convergent evolution [4,5,6], and coevolution [7,8,9]. The mouthparts of adult flies (Diptera, +150,000 species) are no exception and exhibit adaptations for feeding on a wide variety of liquid foods, including nectar, blood, sweat, and fluids on carrion, dung, and rotting fruit [1,10,11]. These diverse feeding habits are matched by a sundry of mouthpart structures and configurations for fluid uptake, including mouthparts modified for piercing-sucking by blood-feeding mosquitoes (Culicidae) [12,13], accessing nectar from long floral tubes by long-proboscis flies (Nemestrinidae) [14], and sponging-sucking on liquid films by house flies (Muscidae), blow flies (Calliphoridae), hover flies (Syrphidae), and other dipteran groups [1,2].

The structural organization of mouthparts adapted for sponging-sucking consists of a proximal rostrum, haustellum, and distal labellum [1,15,16]. The labellum is comprised of two lobes that can open and close; when open, a series of pseudotracheae are revealed [17,18] which provide capillary action [6]. An oral opening near the center of the labellum leads to the food canal for fluid transport to the head. Some dipteran mouthparts have cuticular projections, the prestomal teeth, that line the medial edges of the labellum near the oral opening and rasp surfaces to expose fluids when feeding [19,20,21].

Prestomal teeth morphology has been reported for several dipteran species, particularly those of medical importance, including the tsetse fly, *Glossina pallidipes* (Glossinidae) [22], and representatives of Calliphoridae [23], Muscidae [19,20,24], and Sarcophagidae [21,25]; however, material properties such as hardness, elastic modulus, the extent of sclerotization, and the possible presence of inorganic elements in the cuticle are unknown. Here, we study the material properties of the cuticle of prestomal teeth and combine these results with morphology to better understand how prestomal teeth relate to feeding habits. We hypothesized that species that frequent food sources that require rasping, such as those that must excoriate tissues for fluids, will have larger and harder prestomal teeth with higher concentrations of inorganic elements when compared to species that feed almost exclusively on exposed fluids.

## 2. Materials and Methods

### 2.1. Species

We chose 11 brachyceran fly species that represent a range of feeding habits. The transverse-banded flower fly, *Eristalis transversa* (Wiedemann, 1830) (Syrphidae), black soldier fly, *Hermetia illucens* (L., 1758) (Stratiomyidae), and bristle fly, *Archytas metallicus* (Robineau-Desvoidy, 1830) (Tachinidae) were used as representative species that feed primarily on nectar (referred to hereafter as flower visitors). The cluster fly, *Pollenia pediculata* (Macquart, 1834) (Polleniidae), bird blow fly, *Protocalliphora azurea* (Fallén, 1817) (Calliphoridae), blue bottle fly, *Calliphora vomitoria* (L., 1758) (Calliphoridae), green bottle fly, *Lucilia sericata* (Meigen, 1826) (Calliphoridae), and grey flesh fly, *Sarcophaga bullata* (Parker, 1916) (Sarcophagidae) were used as generalist species that frequently visit flowers for nectar (generalist-flower visitors). The house fly, *Musca domestica* (L., 1758 (Muscidae), was used as a representative generalist species that frequently feeds on animal matter, including carrion and dung (generalist-nonflower visitor). The yellow dung fly, *Scathophaga stercoraria* (L., 1758) (Scathophagidae) frequently feeds on other dipteran species and was used as a representative insectivorous species. The stable fly, *Stomoxys calcitrans* (L., 1758) (Muscidae), a pest of domesticated animals, was used as a representative blood feeder. All individuals were collected in North Canton, OH, USA, from June–August 2021, except for individuals *Sa. bullata* that were ordered as pupae from the Carolina Biological Supply Company (Burlington, NC, USA), *St. calcitrans* were obtained from colonies at the University of Florida and Texas A&M, and *M. domestica* and *C. vomitoria* were ordered as pupae from Josh’s Frogs (Owosso, MI, USA). All individuals had their mouthparts cleaned in dH_2_O and were stored in a −80 °C freezer.

### 2.2. Mouthpart Morphology with Scanning Electron Microscopy

Individuals had their labellum removed with a razorblade and placed into a droplet of dH_2_O on a glass cover slip. The dH_2_O caused the lobes of the labellum to spread apart, revealing the pseudotracheae, oral opening, and, if present, the prestomal teeth. A second coverslip was placed on top to flatten the mouthparts and keep the labellum open. A dehydration series was performed (70, 80, 90, 95, and 100% EtOH, 24 h each) by administering EtOH with a dropper between the coverslips. The mouthparts were then critically-point dried (Leica EM CPD300), secured to an aluminum stub with double-sided carbon graphite tape, and sputtercoated (EMS 150 TES) with 10 nm of platinum. The mouthparts were imaged with a scanning electron microscope (SEM) (JEOL 6010LV) at 20 kV and low magnifications (80–200×) to make measurements of the labellum and higher magnifications (>250×) for prestomal teeth measurements.

**Figure 1:**
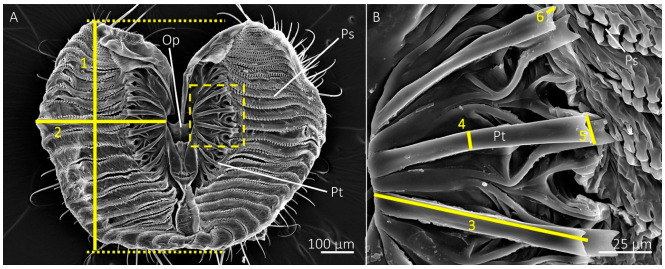
SEM images of the mouthparts of the blue bottle fly, *Calliphora vomitoria*, showing measurements used for morphology. (**A**) The distal region of opened labellum showing the oral opening (Op), pseudotracheae (Ps), and prestomal teeth (Pt). The length (1) and width (2) of a single labellar lobe was measured at their greatest distances, as shown with the solid yellow line. The dotted lines show how length boundaries were determined for accurate measurements. The region in the dotted-lined box is displayed at higher magnification in (**B**) and shows measurements for prestomal teeth, including tooth length (3), width (4), the width of the distal tip (5), and tine length (6).

We measured the length and width of a single lobe of each labellum (measurements 1 and 2 shown in Figure 1a). The lengths were measured by drawing straight lines at the anterior and posterior boundaries of the labellum using Microsoft^®^ PowerPoint, then measuring the distance between these lines (Figure 1a). The widths were measured from the base of a prestomal tooth near the oral opening to the lateral edge of the labellar lobe at its widest point. We also measured the percentage area of a single labellar lobe occupied by prestomal teeth by isolating two images per specimen in Adobe^®^ Photoshop v 23.0 with the eraser tool: one image of a single labellar lobe with the prestomal teeth and another of the prestomal teeth only. The percent area, based on pixel number, of the labellum occupied by prestomal teeth was determined by $\frac{\mathrm{prestomal}\;\mathrm{teeth}\;\mathrm{area}}{\mathrm{labellar}\;\mathrm{lobe}\;\mathrm{area}}$ × 100%. The total number of prestomal teeth and pseudotracheae were recorded for a labellar lobe of each individual.

Measurements of prestomal teeth included length, measured from the base to the tip, and width, measured at the midregion between the base and the tip (measurements 3 and 4 shown in Figure 1b). We also measured tine lengths and the widths of the distal tips of the prestomal teeth (measurements 5 and 6 in Figure 1b). Measurements were acquired for three prestomal teeth for each individual and averaged together to produce representative measurements per individual. All morphology measurements were performed in ImageJ software [26].

### 2.3. Elemental Analysis with Energy Dispersive X-ray Spectroscopy

Individuals studied with SEM had the elemental composition of their prestomal teeth and labellum analyzed with energy dispersive X-ray spectroscopy (EDS) (X-Max50, Oxford Instruments) and Aztec software. The elemental composition was studied with the point and ID tool at three locations per individual: between the pseudotracheae on the labellum (Region 1), the distal tip of a prestomal tooth (Region 2), and at the base of a prestomal tooth (Region 3) (20 kV, spot size of 61–63, magnification of 500×). The percentage weight for each detected element was recorded, but only the most common inorganic elements were used for data analysis.

### 2.4. Studies of Prestomal Teeth and Labellum Material Properties Using Confocal Microscopy

The labellum and prestomal teeth of *M. domestica*, *Sc. Stercoraria*, *St. calcitrans*, and *Sa. Bullata* were used to assess the extent of sclerotization and the possible presence of the elastomeric protein resilin. The labellum was removed with a razorblade and placed into a droplet of glycerol on a glass slide, which was then manipulated with insect pins to expose the prestomal teeth. A high-precision cover slip (12 mm diameter, thickness no. 1.5 H, Neuvitro Corporation, USA) was placed on top. The autofluorescence of mouthpart structures was studied with an Olympus IX81 inverted confocal microscope and the CY3 (531–540 nm excitation, 593–640 nm emission), DAPI (377–360 nm excitation, 447–460 nm emission), and GFP (469–535 nm excitation, 525–639 nm emission) filters. Mouthparts were imaged with autoexposure settings and a 15-slice Z-stack in Olympus cellSens software. Structures that appeared red indicated a high level of sclerotization, green indicated weak levels of sclerotization, and structures that had blue autofluorescence indicated the presence of resilin [27].

### 2.5. Nanoindentation

The labellum of individuals of *Sa. bullata*, *Sc. stercoraria*, and *St. calcitrans* were removed with a razorblade and placed into a droplet of dH_2_O on a glass slide. Insect pins and a razorblade were used to remove the labellar tissue from the prestomal teeth, then the cleaned prestomal teeth were placed into a new droplet of dH_2_O on a glass slide. The prestomal teeth were manipulated with an insect pin so that the anterior surface of the teeth was exposed, and the slide was placed briefly on a hot plate to evaporate any remaining dH_2_O.

Nanoindentation measurements were performed using a Bruker Hysitron TI Premier nanoindenter, configured with a Ti-0045 cono-spherical probe (90° cone angle, 5 µm tip radius, diamond coated). All measurements were set up, performed, and analyzed through the TriboScan v9.6 software. It was confirmed before the experiments that the actual shape of the indenter probe was in good agreement with the default area-function of a spherical probe, $A\;=-\pi \cdot h^{2}+2\pi R\cdot h$, where *A* is the cross-sectional area, *h* is the indentation depth, and *R* is the tip radius of the probe. The specimens were then measured with load-controlled quasi-static indentation tests using a standard trapezoidal loading function (5 s loading, followed by 2 s dwell, and lastly 5 s unloading). The maximum load was set at 2000 µN for all measurements. The resulting force-displacement curves were then analyzed by the built-in analysis routine, which is based on the Oliver-Pharr Method [28], and the reduced modulus, as well as the hardness, were directly obtained from the TriboScan software. The reduced modulus was then converted to elastic modulus with the assumption that the Poisson’s ratio of the prestomal teeth is 0.3 [29,30].

### 2.6. Statistics

Analysis of variance (ANOVA) was used to determine significant differences (*p* < 0.05) in the morphological measurements and EDS data among and within species. ANOVA also was used to compare the hardness and elastic modulus measurements among tested species. Significant differences in means were ranked using a Student’s *t*-test. A linear discriminant analysis was used to assess a classification system with the EDS data for each studied region among species. A hierarchical clustering analysis (Ward’s method with standardized data) was used to concurrently evaluate the morphology data and the EDS data to create a dendrogram to determine if the 11 studied species are grouped according to feeding habits. All statistics were performed in JMP v16 software.

## 3. Results

### 3.1. Labellum Morphology

Labellar lobe length differed significantly among species (df = 10, 44; F = 44.5082; *p* < 0.0001) and was generally longest among the flower-visiting fly species (Table 1). For example, *H. illucens* had the longest labellar lobe, followed by *E. transversa*, another flower visitor. The shortest length was observed on the blood feeder *St. calcitrans*, followed by the flower visitor *A. metallicus*, and the insectivorous *Sc. stercoraria*. Labellar lobe width showed a similar pattern (df = 10, 44; F = 24.7716; *p* < 0.0001) with *H. illucens* having the widest labellar lobe and *St. calcitrans* having the smallest (Table 1). The generalist-flower visiting species and *M. domestica*, a generalist-nonflower feeder, had relatively similar proboscis lengths and widths.

The number of pseudotracheae differed significantly among species (df = 10, 44; F = 269.9875; *p* < 0.0001), with *H. illucens*, *E. transversa*, and *M. domestica* having the most pseudotracheae. The generalist-flower visitors also had a high number of pseudotracheae; however, *Sc. stercoraria* had a low number of pseudotracheae and no pseudotracheae were observed on the labellum of *St. calcitrans* (Table 1).

The percentage area of the labellum occupied by prestomal teeth differed significantly among species (df = 10, 44; F = 90.8249; *p* < 0.0001) (Table 1). The blood feeder *St. calcitrans* had almost 40% of their labellum occupied by prestomal teeth and the insectivorous *Sc. stercoraria* followed with over 18%. The generalist-flower visiting species had approximately 10% of the labellum covered by prestomal teeth, except for *Po. vagabunda*, which only had approximately 3% and was similar to *M. domestica*.

### 3.2. Prestomal Teeth Morphology

**Figure 2:**
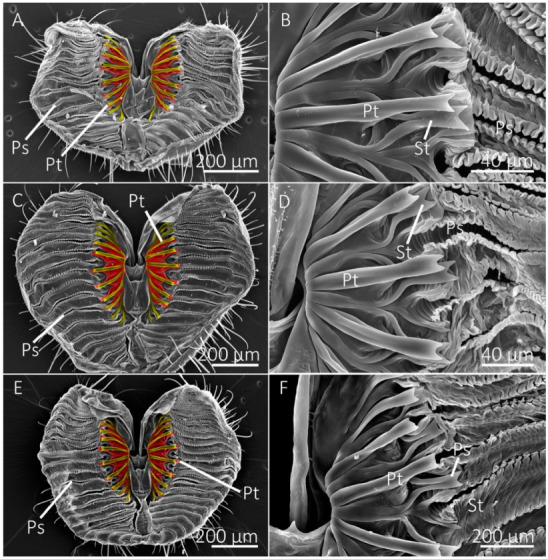
SEM images of the labellum and prestomal teeth of the generalist-flower visiting species. (**A**) The labellum of the green bottle fly, *Lucilia sericata*, showing pseudotracheae (Ps) and prestomal teeth (Pt), shown in (**B**) at a higher magnification. (**C**,**D**) Labellum and prestomal teeth of *Protocalliphora azurea*. (**E**,**F**) Labellum and prestomal teeth of *Calliphora vomitoria*. (**A**,**C**,**E**) Primary prestomal teeth are false-colored yellow and secondary prestomal teeth (St in **B**,**D**,**F**) are false-colored red.

**Figure 3:**
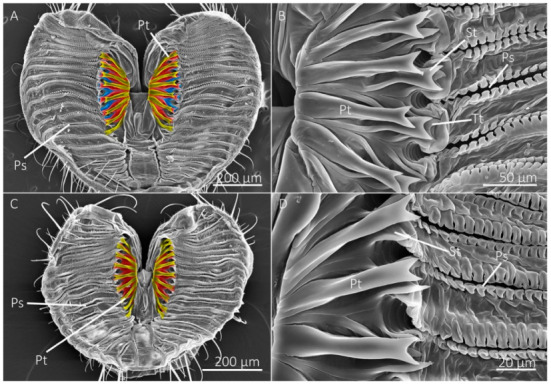
SEM images of the labellum and prestomal teeth of generalist-flower visiting species. (**A**,**B**) The labellum of *Sarcophaga bullata* shows prestomal teeth (Pt) and pseudotracheae (Ps). The primary prestomal teeth are false-colored in yellow, secondary teeth are red (St in (**B**)), and tertiary prestomal teeth (third row of teeth) are shown in blue (Tt in (**B**)). (**C**,**D**) The labellum and prestomal teeth of *Pollenia pediculata*.

**Figure 4:**
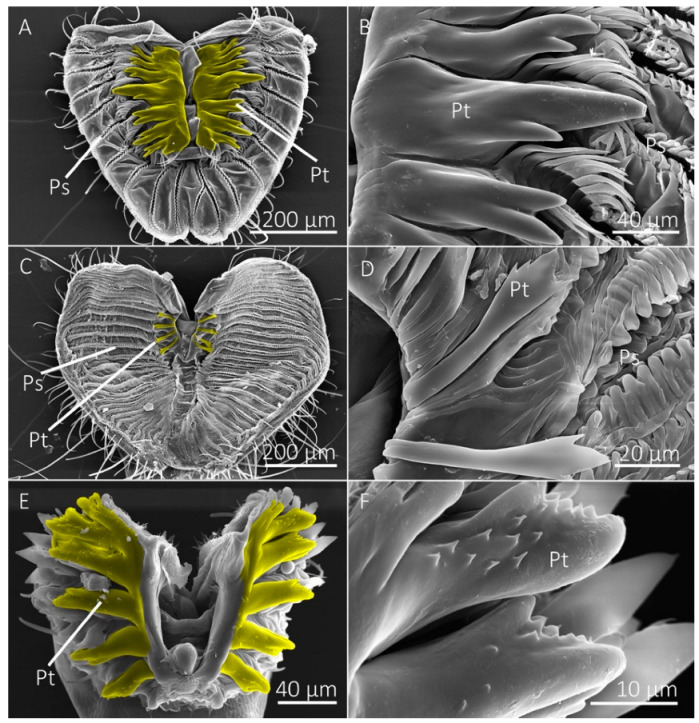
SEM images of labellum and prestomal teeth among flies of different feeding habits. (**A**) The labellum of *Scathophaga stercoraria*, an insectivorous species, with large prestomal teeth (Pt) and pseudotracheae (Ps), is also shown at higher magnifications in (**B**). (**C**,**D**) The labellum and prestomal teeth of *Musca domestica*, a generalist-nonflower visitor. (**E**,**F**) The labellum and prestomal teeth of *Stomoxys calcitrans*, which lacks pseudotracheae. The prestomal teeth of *St. calcitrans* have smaller spines on them. The species shown here only have a single row of prestomal teeth (false-colored yellow in (**A**,**C**,**E**)).

**Figure 5:**
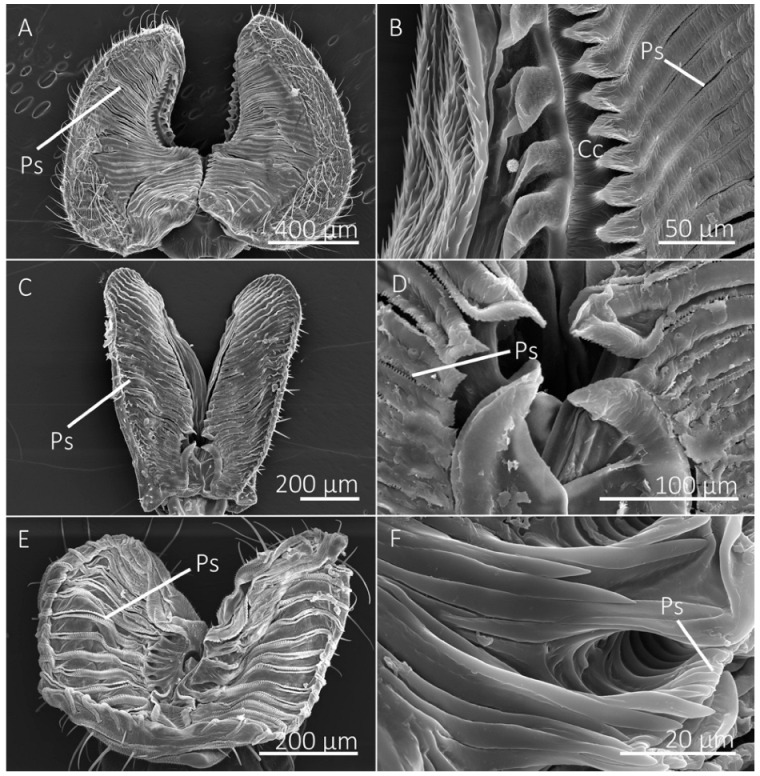
SEM images of the labellum of flower visiting fly species. (**A**,**B**) The labellum of *Hermetia illucens* shows the pseudotracheae (Ps), which lead to a collecting conduit (Cc) on the medial sides of the labellar lobes. (**C**,**D**) Labellum and pseudotracheae of *Eristalis transversa*. (**E**,**F**) The labellum and pseudotracheae of *Archytas metallicus* have cuticular projections near the oral opening.

The number of prestomal teeth differed significantly among species (df = 10, 44; F = 127.5146; *p* < 0.0001) (Table 2). The generalist-flower visiting species had the highest number of prestomal teeth (Figure 2 and Figure 3), which was followed by *Sc. stercoraria*, *St. calcitrans*, and *M. domestica* (Figure 4). The flower visiting species lacked prestomal teeth (Figure 5).

The length of the prestomal teeth also differed significantly among species (df = 7, 29; F = 58.2110; *p* < 0.0001), with *Sc. stercoraria* having the longest teeth (Table 2). The generalist-flower visiting species had teeth of similar lengths, which were longer than those of *M. domestica* and *St. calcitrans*. Measurements of prestomal teeth widths generally showed a similar pattern (df = 7, 29; F = 138.8251; *p* < 0.0001) with *Sc. stercoraria* having the widest teeth, followed by *St. calcitrans*, and the generalist-flower visiting species (Table 2).

Tine length differed significantly among species (df = 7, 29; F = 105.3093; *p* < 0.0001), as did the width of the distal tip of the prestomal teeth (df = 7, 29; F = 15.2437; *p* < 0.0001) (Table 2). The insectivorous *Sc. stercoraria* had the longest tine length, followed by *M. domestica* and *St. calcitrans* (Figure 4). The generalist-flower visiting species had the shortest prestomal teeth (Figure 2 and Figure 3). A similar pattern was observed in the widths of the distal tips of prestomal teeth, however, they were significantly wider in *Sa. bullata* than the other generalist-flower visiting species. The number of tines on each prestomal tooth also differed among species (df = 7, 29; F = 19.2450; *p* < 0.0001) (Table 2). The prestomal teeth of *St. calcitrans* consisted of large lobes with several spike-like tines and smaller spike-like projections near the midregion and distal region (Figure 4). The number of tines also was large for *M. domestica* (Figure 4) and their overall shape was broader in their distal regions. All remaining species had prestomal teeth that were primarily bifurcated (Figure 2 and Figure 3).

### 3.3. Differences in Elemental Composition among Regions within Each Species

**Figure 6:**
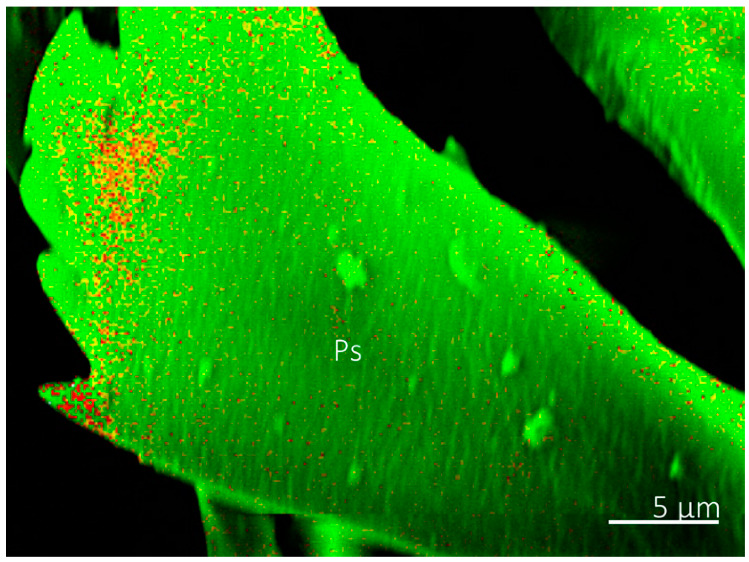
Energy dispersive x-ray spectroscopy image of a prestomal tooth of *Musca domestica*. The image shows that the prestomal tooth (Ps) has Zn (red), Si (yellow), and C (green).

EDS revealed an array of elements in the mouthpart cuticle, including C, O, Na, S, Cl, K, Ca, Al, Fe, Mg, P, Si, and Zn. We focused the remainder of the analysis only on the most common inorganic elements that might augment cuticular properties, including Na, S, Cl, K, Ca, and Mg. Although not included in the further analysis because of their inconsistency in presence within and among species, Fe was found in the prestomal teeth of some individuals of *M. domestica*, *St. calcitrans*, and *Sc. stercoraria,* and Zn was found in *St. calcitrans* and *M. domestica* (Figure 6).

The elemental composition among the three studied mouthpart regions differed within species (Table 3, Supplementary Table S1). The prestomal teeth of *C. vomitoria* had significant differences in the concentrations of Na, S, Ca, Mg, and P; all of these elements were significantly higher in Region 1, the region measured between the pseudotracheae on the labellum. Although present, there were no significant differences among regions for Cl and K. A similar pattern was found in *L. sericata* for concentrations of K and P, but no significant differences among regions for Na, S, Cl, Ca, and Mg. There were significant differences in concentrations of S, Cl, K, Ca, Mg, and P among regions for *Pr. azurea*, with higher concentrations in Region 1, except for Cl, which was highest in Region 3 (base of prestomal teeth). Na, though present, did not differ significantly among regions. There were significant differences in concentrations of Na, Cl, and Mg for *Sa. bullata*: Na and Mg concentrations were highest in Region 1, but Cl was highest in Region 3. Elements that did not differ significantly among regions for *Sa. bullata* were S, K, Ca, and P. Of the generalist-flower visiting species, *Po. vagabunda* had the lowest diversity of elements: Na, K, and Ca were not detected. The only element that significantly differed in concentrations among regions was Mg, which was highest in Region 1. Although present, there were no significant differences among regions for S, Cl, and P (Table 3).

The generalist-nonflower feeder, *M. domestica*, had a large diversity of elements in the mouthpart cuticle (Table 3). The only element that showed significant differences among regions, however, was Cl, which was highest in Region 3. The elements Na, S, K, Ca, Mg, and P also were present (Table 3, Supplementary Table S1).

The insectivorous *Sc. stercoraria* displayed significant differences in elemental composition among mouthpart regions for S, Cl, and P (Table 3), where S and P were in the highest concentrations in Region 1, but Cl was highest in Region 3. Other elements present included Na, Ca, and Mg. The element K was not detected in this species (Table 3, Supplementary Table S1).

Elemental analysis for the blood feeder *St. calcitrans* indicated that only S significantly differed among regions and was highest in Region 1. Although no pseudotracheae were observed, measurements for Region 1 were still recorded on the fleshy portion of the labellum. The elements Na and Mg were not recorded for this species and other detected elements (Cl, K, Ca, and P) did not significantly differ in concentrations among regions (Table 3, Supplementary Table S1).

### 3.4. Differences in Elemental Composition within Tested Regions among Species

In Region 1, Na significantly differed among species, with the highest concentrations in *C. vomitoria*, followed by *Sa. bullata* and *Pr. azurea*, and the other generalist-flower visiting species (Table 3). Concentrations of Cl also differed among species, with significantly higher amounts in the blood feeder *St. calcitrans*. Although present, S, K, Ca, Mg, and P did not differ significantly among species (Table 3, Supplementary Table S2).

Regions 2 and 3, which correspond to measurements on the distal region and base of the prestomal teeth, respectively, also revealed patterns in elemental composition (Table 3). There were significant differences in Region 2 among species for Na, S, Cl, K, Ca, and P. In general, the generalist-flower visiting species had the highest concentrations of Na; however, *M. domestica* had the highest or nearly the highest concentrations of S, Cl, Ca, and P (Table 3). Mg did not differ significantly in Region 2 among species.

There also were differences in the elemental composition of Region 3 among species (Table 3, Supplementary Table S2). The generalist-flower visitors had significantly higher concentrations of Na. Although present, there were no significant differences among species for the elements S, Cl, K, Ca, Mg, and P.

### 3.5. Species Classification and Clustering with EDS and Morphology Measurements

A linear discriminant analysis was performed on the EDS data for each of the studied regions among species and revealed a classification system that primarily assembled each species separately (Table 4). Of the three studied mouthpart regions, the linear discriminant analysis had the most inconsistencies in Region 1. All species had incorrectly grouped individuals, except for *Po. vagabunda*, which was 100% correctly categorized. There was an accurate classification for *C. vomitoria* (80%), with one individual that was incorrectly classified as *Sa. bullata*. Only 50% of *L. sericata* were correctly classified and the other individuals were classified as either *Pr. azurea*, *Sa. bullata*, or *Sc. stercoraria*. Only one individual of *M. domestica* was correctly categorized and the other individuals were classified as *Po. vagabunda*, *Pr. azurea*, *Sa. bullata*, and two individuals classified as *Sc. stercoraria*. Only 33.33% of *Pr. azurea* were correctly classified and the remaining individuals were incorrectly classified as *Po. vagabunda*, *Sa. bullata*, and *Sc. stercoraria*. There was a relatively accurate classification of *Sa. bullata* (60%), but the other individuals were classified as *C. vomitoria* and *Pr. azurea*. Nearly all individuals of *Sc. stercoraria* were classified correctly (75%) and the remaining individuals were incorrectly classified as *Pr. azurea*. Only 33.33% of the individuals of *St. calcitrans* were correctly categorized with the remaining individuals incorrectly classified as *Sc. stercoraria* (Table 4).

The linear discriminant analysis revealed a more accurate classification system for Regions 1 and 2; however, the distal region of the prestomal teeth (Region 2) generally was the most accurate (Table 4). For example, 83.33% of *M. domestica* were correctly classified in Region 2 with the remaining individuals classified as *Sa. bullata*, whereas Region 3 had only 33.33% correctly classified, with 33.33% classified as *L. sericata* and 33.33% as *Po. vagabunda*. Most individuals of *C. vomitoria* were correctly classified in Region 2 and the other individuals were classified as *Sa. bullata* and *Sc. stercoraria*; however, 83.33% were correctly classified in Region 3 with one individual incorrectly classified as *Po. vagabunda*. There were similar patterns of classification for *L. sericata* in Regions 2 and 3, with 50% correctly classified in Region 2 and the other individuals incorrectly classified as either *Pr. azurea*, *Sc. stercoraria*, and *St. calcitrans*, and 50% correctly classified in Region 3, with the other individuals incorrectly classified as *C. vomitoria* and *Po. vagabunda*. Individuals of *Po. vagabunda* were classified well in both regions, with some individuals incorrectly classified as *Pr. azurea* in Region 2 and *L. sericata* in Region 3. Most individuals of *Pr. azurea* were correctly classified in Region 2 (83.33%) with one individual incorrectly classified as Sc. stercoraria, whereas there was only one individual correctly classified in Region 3 and the remaining individuals classified as C. vomitoria, *L. sericata*, *Po. vagabunda*, and *St. calcitrans*. In Region 2, 80% of *Sa. bullata* were correctly categorized and one individual was incorrectly classified as *C. vomitoria*, and 60% were correctly classified in Region 3 with the other 40% incorrectly classified as *Sa. bullata*. The insectivorous *Sc. stercoraria* had 100% of individuals correctly classified in Region 2 and 75% correctly classified in Region 3, with the other 25% incorrectly classified as *St. calcitrans*. The blood feeder *St. calcitrans* had 66.66% individuals correctly classified in Regions 2 and none correctly classified in Region 3, with the other individuals incorrectly classified as *Pr. azurea* in Region 2 and *L. sericata* and *Sc. stercoraria* in Region 3 (Table 4).

**Figure 7:**
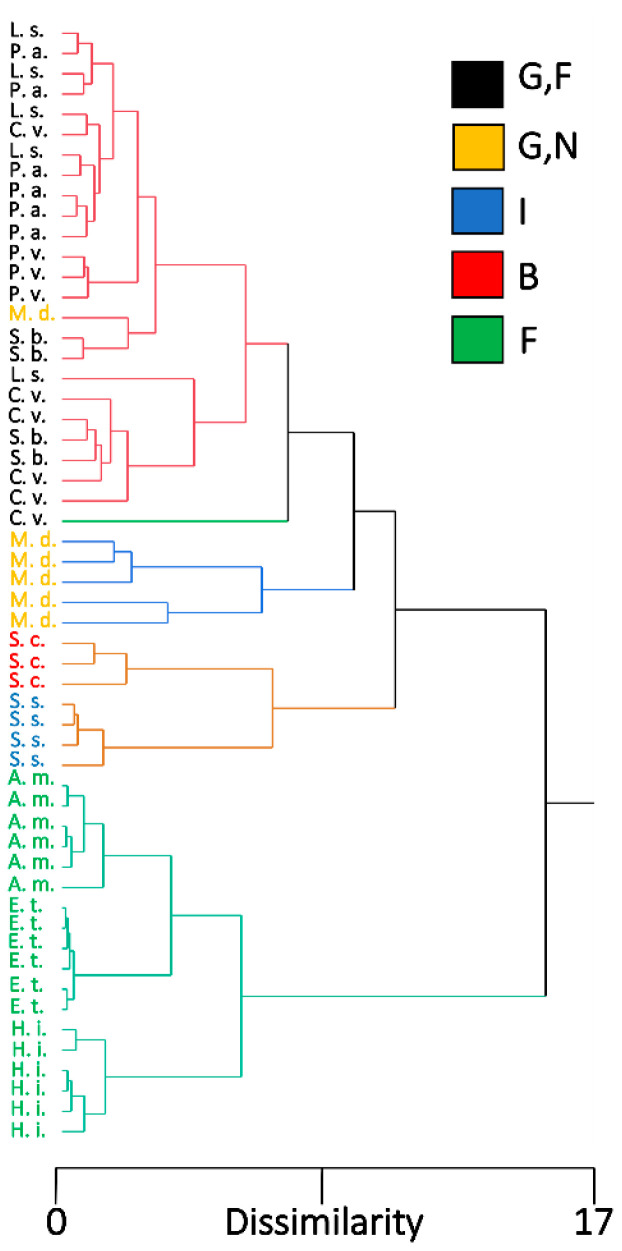
Dendrogram from a hierarchical cluster analysis showing the pattern of individuals among different fly species based on morphological measurements and EDS measurements from Region 2. Five clusters were assigned to represent different feeding habits. Fly species are color-coded according to their feeding habits, G,F (black) are flies with generalist-flower visiting feeding habits, G,N (yellow) is generalist-nonflower feeders, I (blue) is insectivorous, B (red) is blood feeders, and F (green) is flower visitors. L. s. = *Lucilia sericata*, M. d. = *Musca domestica*, C. v. = *Calliphora vomitoria*, P. a. = *Protocalliphora azurea*, P. p. = *Pollenia pediculate*, S. b. = *Sarcophaga bullata*, S. s. = *Scathophaga stercoraria*, S. c. = *Stomoxys calcitrans*, A. m. = *Archytas metallicus*, E. t. = *Eristalis transversa*, and H. i. = *Hermetia illucens*.

We combined EDS measurements from Region 2, because of the generally more accurate classification system from the linear discriminant analysis, with the morphological measurements for hierarchical cluster analysis and chose to use five clusters to represent the five feeding habits. The dendrogram produced from the hierarchical cluster analysis showed that the species were grouped according to feeding habits (Figure 7). All flower visitors produced a cluster, with additional subclusters representing species groups. Although the blood-feeder *St. calcitrans* and the insectivorous *Sc. stercoraria* shared a cluster, they did produce separate smaller clusters representing their different feeding habits. A cluster was produced that grouped the generalist species together; however, *M. domestica* was grouped into a smaller cluster that was separate from the generalist-flower visitors. All individuals of the generalist-flower visiting species created a cluster, except for one individual of *C. vomitoria* that produced its own cluster. Individuals did not produce species-specific smaller clusters within the cluster of generalist-flower visitors (Figure 7).

### 3.6. Material Properties of the Prestomal Teeth and Labellum

**Figure 8:**
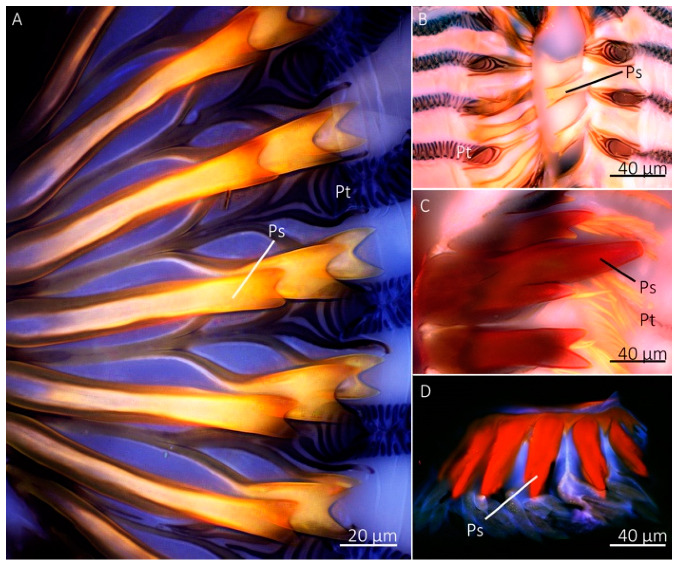
Confocal microscopy images of the prestomal teeth and labellum of different fly species. The prestomal teeth of *Sarcophaga bullata* (**A**), *Musca domestica* (**B**), *Scathophaga stercoraria* (**C**), and *Stomoxys calcitrans* (**D**) revealed red autofluorescence. The labellum of all species appeared blue.

Confocal microscopy revealed a similar pattern in autofluorescence of the labellum and prestomal teeth among *Sa. bullata*, *M. domestica*, *Sc. stercoraria*, and *St. calcitrans* (Figure 8). We used these species because they represent the different feeding habits among those groups with prestomal teeth. The prestomal teeth appeared red in all studied species, indicating a high level of sclerotization. The labellum, however, was blue, indicating the presence of the elastomeric protein resilin. No green, which indicates an intermediate level of sclerotization, was observed when all three channels were combined into a single image. The prestomal teeth of *Sc. stercoraria* and *St. calcitrans* appeared a deeper red than the other species, possibly due to their increased thickness and reduced translucency.

**Figure 9:**
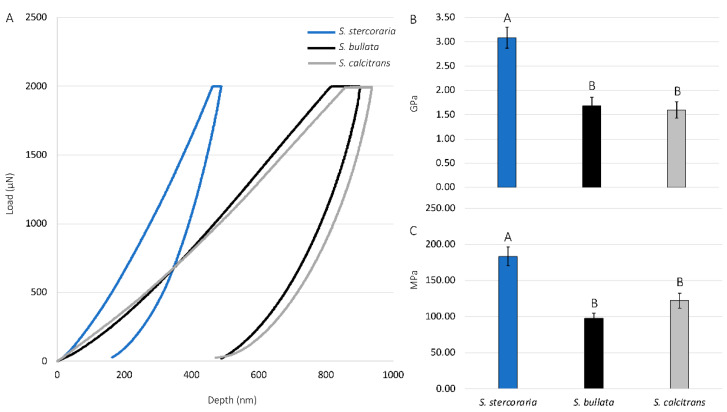
Force curves, modulus, and hardness values of prestomal teeth for three dipteran species. (**A**) Representative force curves for *Scathophaga stercoraria*, *Sarcophaga bullata*, and *Stomoxys calcitrans*, as determined by nanoindentation using a maximum force load of 2000 µN. Elastic modulus values (**B**) and hardness values (**C**) for dipteran species. The modulus and hardness of the prestomal teeth were significantly higher in *Sc. stercoraria* (significant differences shown with capital letters). The other tested species had similar values.

We further studied the material properties of the prestomal teeth by quantifying the elastic modulus and hardness using nanoindentation (Figure 9). There were significant differences in both the elastic modulus and hardness of the prestomal teeth among species (df = 2, 24; F = 18.1392; *p* < 0.0001 for the elastic modulus, df = 2, 24; F = 17.7052, *p* < 0.0001 for hardness). Prestomal teeth of the insectivorous *Sc. stercoraria* exhibit the highest elastic modulus and hardness. The hardness and elastic modulus values for Sa. bullata and *St. calcitrans* did not significantly differ from each other. We did not perform nanoindentation analysis on the prestomal teeth of *M. domestica* due to difficulty removing them from the labellar tissue.

## 4. Discussion

Diptera is one of the most successful groups of animals from both an evolutionary and ecological perspective [31,32,33], which likely relates to their ability to exploit a wide range of nutritional fluids. Our hypothesis was partially supported in that the morphology and certain aspects of the material properties of the prestomal teeth relate to feeding habits, but our hypothesis that the prestomal teeth have a high concentration of inorganic elements was not supported. In addition, our results revealed several themes and adaptations for feeding that are further described here.

### 4.1. Labellum and Prestomal Teeth Morphology Relate to Feeding Habits

Diptera with sponging-sucking mouthparts have a structural configuration that allows them to exploit liquid films. The ability to feed on liquid films is linked to the pseudotracheae, as these structures provide capillary action to move small amounts of liquids from surfaces into their conduits for subsequent uptake at the oral opening [6]. Prestomal teeth, when present, can increase liquid availability by rasping surfaces [19,20,21]. Although the prestomal teeth and the pseudotracheae both assist in fluid feeding, there is a tradeoff—large prestomal teeth could block or hinder pseudotracheae functionality. This tradeoff agrees with our interpretation of structure-function relationships and how they pertain to feeding habits.

Diptera that primarily visit flowers to feed on nectar lack prestomal teeth and instead have a mouthpart organization that prioritizes fluid collection via capillary action (Table 1). The mouthpart morphology of the flower visiting *H. illucens* and *E. transversa*, for example, consisted of numerous pseudotracheae that lead to a larger collecting conduit on the medial side of the labellum, which then leads to the oral opening (Figure 5). This hierarchical arrangement of the pseudotracheae and collecting conduits provides a method to overcome the physical limitations that would be associated with feeding on liquid films on porous surfaces, as described by the limiting pore-size hypothesis [6,34]. The lack of prestomal teeth, however, indicates that these species are unable or at least inefficient at exposing fluids beyond what is already available on the wetted substrate surface. We consider these species as specialists for feeding on liquid films that are already exposed. The flower visiting tachinid fly, *A. metallicus*, also had collecting conduits; however, they were less pronounced than the other flower visitors and several pseudotracheae led directly to the oral opening rather than a collecting conduit. Although *A. metallicus* lacked prestomal teeth, there were several small cuticular projections between the pseudotracheae near the oral opening, perhaps vestigial prestomal teeth, and given their small size we suspect they do not play a role in rasping surfaces (Figure 5).

The generalist-flower visiting species also had a large labellum and numerous pseudotracheae, but their labellum was generally smaller and the pseudotracheae were fewer in number than the flower visitors (Table 1). Generalist-flower visiting species also had prestomal teeth of similar sizes and dimensions (Table 2, Figure 2 and Figure 3). The structural configuration of their mouthparts would allow access to films of nectar and other food sources where fluid acquisition requires rasping; however, the extent of rasping capabilities might be minimal as the prestomal teeth occupy only a small percentage of the labellum surface (Table 1). A similar mouthpart configuration was observed in *M. domestica*, the generalist-nonflower visitor (Figure 4), but this species had a higher number of pseudotracheae and smaller prestomal teeth (Table 1 and Table 2).

The insectivorous *Sc. stercoraria* and the blood feeder *St. calcitrans* had a mouthpart morphology that represented the opposite extreme in the pseudotracheae and prestomal teeth tradeoff (Figure 4). These species have lower numbers of prestomal teeth than the generalist species and a labellum that was primarily occupied by prestomal teeth (Table 1 and Table 2). The pseudotracheae of *Sc. stercoraria* were widely spaced and they had the largest prestomal teeth. This mouthpart configuration suggests the ability to feed on liquid films, but that ability would be reduced because of the large prestomal teeth, and there is likely a greater emphasis on feeding on pools of fluids, such as hemolymph, from their insect prey. The emphasis on using prestomal teeth to feed on pools of fluids also is shown in *St. calcitrans* where there are no pseudotracheae, thus their mouthparts lack the ability to use capillary action to feed on liquid films. Although this species had the smallest labellum, they had the highest percentage of their labellum dedicated to prestomal teeth (Table 1). Both species rely on the prestomal teeth to puncture or lacerate animal tissue to access pools of nutritional fluids.

### 4.2. Prestomal Teeth Material Properties in Relation to Feeding Habits

We hypothesized that the material properties of the prestomal teeth, including the presence of inorganic elements, the extent of sclerotization, hardness, and elastic modulus relate to feeding habits; however, not all of these were supported. In particular, we expected the prestomal teeth of *Sc. stercoraria* and *St. calcitrans* to have higher amounts of inorganic elements than species of other feeding habits, but this was not observed (Table 3). Metal ions and other inorganic elements are known to enhance the material properties of insect cuticles, particularly on structures that rasp or pierce hard substrates [35,36,37,38]. Here, the studied cuticular elements were in low concentrations (mostly <1%) for all species. The generalists, with the exception of *Po. vagabunda* had the highest amounts of inorganic elements, and low concentrations were found in *Sc. stercoraria* and *St. calcitrans*. It is worth noting, however, that Cl was generally present in the highest quantities in the prestomal teeth (Regions 1 and 2, Table 3). Halogens, such as Cl, play a role in providing functionality to transition metals, when present, in arthropod cuticles [39,40]. Even though there are low concentrations of inorganic elements in the prestomal teeth, their presence, along with Cl, suggests that they might play some role in augmenting the cuticle, either by increasing hardness or in some other way, such as reducing wear due to friction forces and being less prone to breakage.

The inorganic elements in the adult cuticle are likely sequestered from the environment, probably through larval food sources; this is when food that contains the inorganic elements would be ingested prior to the final molt when the new cuticle is formed with the integrated inorganic elements. The species studied here that have prestomal teeth feed on a variety of foods, such as larvae, including coprophagous feeding habits of *Sc. stercoraria*, *St. calcitrans*, and *M. domestica* [41,42], sarcosaprophagy by *C. vomitoria*, *L. sericata*, and *Sa. bullata* [43,44,45], feeding on the blood of young birds by *Pr. azurea* [46], and earthworm-feeding by *P. pediculata* [47]. The lack of larger quantities of inorganic elements in the prestomal teeth suggests that either the studied species have larval diets that lack inorganic elements in significant portions or perhaps these species lack the ability to sequester them and allocate them to specific structures, such as the prestomal teeth, but this requires further study.

Although there was a lack of inorganic elements in the cuticle of prestomal teeth, confocal microscopy and nanoindentation revealed that the teeth are hard. Confocal microscopy has been used on several arthropods to assess their material properties [3,27,48,49,50]. Here, the labellum of all studied species had blue autofluorescence, an indicator of the elastomeric protein resilin. The presence of resilin in the labellum is unsurprising as it is expected that this structure would have high elasticity to allow it to mold to wetted rough surfaces for feeding. The prestomal teeth had red autofluorescence indicating a high level of sclerotization. We envision a scenario where Diptera place the elastic labellum on a wetted surface where it molds to the rough surface and forms a seal that assists in creating a vacuum for fluid uptake, while small movements of the labellum cause the hard prestomal teeth to move back and forth, rasping surfaces to increase liquid availability.

The use of nanoindentation confirmed the hardness and quantified the elastic modulus of the prestomal teeth. Hardness is often defined as a material’s resistance to permanent deformation at a particular force load during indentation, and elastic modulus is defined as the ratio of stress to strain during the deformation of the material [51,52,53]. Nanoindentation has been used to assess the hardness and elastic modulus for a range of insect species, including species of beetles (Coleoptera) [54,55,56,57], bed bugs (Hemiptera) [58], locusts (Orthoptera) [29,59], and others [53]. Here, *Sc. stercoraria* had the highest hardness and elasticity values when compared to *Sa. bullata* and *St. calcitrans* (Figure 9), which corresponds to their feeding habits. The insectivorous *Sc. stercoraria* is tasked with having to pierce the hard cuticle of other insects to feed, whereas *St. calcitrans* uses their prestomal teeth to pierce vertebrate tissue. Prior studies, through using a variety of techniques, have revealed differences in the forces required for piercing a range of tissues: low for pig tissue (<2 N), which requires cutting by mouthparts of St. calcitrans, and much higher for weevil cuticle (> 45 N) that *Sc. stercoraria* might pierce for hemolymph acquisition [57,60]. The high hardness and modulus values for the prestomal teeth of *Sc. stercoraria* are similar to other reported values for insects (e.g., 2.23 GPa for the elytra of the dung beetle, *Geotrupes stercorarius*) [53,57] and have the material properties necessary for piercing insect cuticle. The values reported here, however, might not relate well with the values of living and actively feeding individuals, as the material properties of insect cuticles change when wetted [61]. As a comparison, the reported values here indicate that the hardness and modulus values are similar to those of polycarbonate [62], a strong polymer.

## 5. Conclusions

The dipteran species used in this study represent a phylogenetically diverse group (eight families) that expresses a wide range of feeding habits. Although there were similarities in the mouthpart ground plan among the studied species, our study revealed that mouthpart morphology and the presence and concentrations of inorganic elements result in an assemblage that clusters species according to their feeding habits (Figure 7), not phylogeny [32]. Finding an assemblage pattern that fits feeding habits rather than phylogeny is not unique among studies of insect mouthparts and provides examples of convergent evolution [5,6,63] and demonstrates the strong selective pressures for certain mouthpart configurations and optimal liquid acquisition abilities from a variety of feeding substrates.

Some of the dipteran species studied here feed on a range of available fluids in the environment, including nectar, and provide important pollinator services. Tachinidae, Syrphidae, and Calliphoridae are well known to serve as pollinators and pollen consumers [1,64,65,66]. Although less is known about the feeding habits of wild *H. illucens* [67], other Stratiomyidae are known to be pollinators and possibly pollen consumers too [68]. The morphology of *H. illucens* examined here and in previous studies [69] indicates that the mouthparts are well adapted for feeding on exposed fluids.

The dipteran species used here were categorized into particular feeding habits; however, many species are opportunistic and will feed on an array of nutritive fluids that might be available in the environment. Given their importance in pollination services and other ecologically important roles, we suggest that future studies employ feeding experiments to better understand how prestomal teeth shape, size, and material properties increase fluid availability on a variety of substrate surfaces.

## Figures and Tables

**Table 1:** Mouthpart measurements (means ± SE) for 11 fly species and results of tests of significance using ANOVA.

Species	Feeding Habits	n	Labellum Length (µm)	Labellum Width (µm)	No. of Pseudotracheae	Percent Area of Prestomal Teeth
*C. vomitoria*	G,F	6	657.56 ± 31.11cde	391.33 ± 11.51bc	20.5 ± 0.81de	9.21 ± 0.41c
*L. sericata*	G,F	5	650.81 ± 34.02de	366.9 ± 21.02cd	20.8 ± 0.37de	10.14 ± 0.8c
*Pr. azurea*	G,F	6	697.12 ± 17.44cd	369.77 ± 19.81cd	21.83 ± 0.48d	9.02 ± 0.39c
*Sa. bullata*	G,F	4	740.47 ± 30.89c	434.19 ± 10.96b	25.75 ± 0.25c	8.66 ± 1.22c
*Po. vagabunda*	G,F	3	594.62 ± 45.78ef	354.91 ± 28.74cd	20 ± 0.58ef	2.89 ± 0.86d
*A. metallicus*	F	6	505.76 ± 28.26f	322.23 ± 30.58de	18.33 ± 0.61f	0 ± 0d
*E. transversa*	F	6	848.11 ± 15.25b	273.82 ± 5.39e	30.33 ± 0.49b	0 ± 0d
*H.illucens*	F	6	988.51 ± 40.02a	569.5 ± 30.47a	36.5 ± 0.22a	0 ± 0d
*M.domestica*	G,N	6	646.77 ± 20.51de	337.48 ± 16.73cd	30.33 ± 0.71b	1.87 ± 0.32d
*St. calcitrans*	B	3	144.80 ± 6.81g	96.53 ± 8.96f	0 ± 0h	38.85 ± 4.54a
*Sc. stercoraria*	I	4	519.14 ± 33.09f	269.39 ± 25.68e	10 ± 0.41g	18.43 ± 2.23b

Lowercase letters indicate significant differences (*p* < 0.05) within columns among species using the Student’s *t*-test. G,F = generalist-flower visitor; F, flower visitor; G,N, generalist-nonflower visitor; B, blood feeder; I, insect feeder.

**Table 2:** Measurements (means ± SE) and characteristics of prestomal teeth for 11 fly species and the results of tests of significance using ANOVA.

Species	n	No. of Teeth	Teeth Length	Teeth Width-Midregion	Teeth Width-Distal Tip	Tine Length	No. of Tine
*C. vomitoria*	6	9 ± 0.37ab	117.66 ± 3.02b	12.89 ± 0.39c	15.76 ± 0.66c	4.81 ± 0.26d	2.56 ± 0.14c
*L. sericata*	5	9.6 ± 0.4a	110.95 ± 2.75b	12.7 ± 0.67c	14.96 ± 1.24cd	7.49 ± 0.98cd	2 ± 0c
*Pr. azurea*	6	9.17 ± 0.48ab	114.41 ± 1.76b	12.77 ± 0.27c	13.27 ± 0.49cd	6.36 ± 0.47d	2 ± 0c
*Sa. bullata*	4	9.5 ± 0.5ab	118.2 ± 4.07b	13.64 ± 0.95c	19.28 ± 1.13b	9.8 ± 1.36bc	2 ± 0c
*Po. vagabunda*	3	8.33 ± 1.2b	65.69 ± 5.27c	9.96 ± 0.29d	11.37 ± 0.94d	6.79 ± 0.84cd	2 ± 0c
*A. metallicus*	6	0 ± 0e	NA	NA	NA	NA	NA
*E. transversa*	6	0 ± 0e	NA	NA	NA	NA	NA
*H.illucens*	6	0 ± 0e	NA	NA	NA	NA	NA
*M.domestica*	6	4.33 ± 0.33d	70.47 ± 3.33c	7.48 ± 0.65e	20.17 ± 1.37b	10.99 ± 1.33b	4.83 ± 0.68b
*St. calcitrans*	3	5.67 ± 0.33c	64.46 ± 3.87c	18.09 ± 1.17b	15.49 ± 0.96c	12.67 ± 0.8b	6.22 ± 0.48a
*Sc. stercoraria*	4	6.75 ± 0.25c	138.14 ± 5.37a	32.5 ± 0.6a	26.03 ± 1.73a	39.58 ± 1.73a	2 ± 0c

Lowercase letters indicate significant differences (*p* < 0.05) within columns among species using the Student’s *t*-test.

**Table 3:** Energy dispersive X-ray spectroscopy measurements (means ± SE) of the most common inorganic elements in the cuticle of prestomal teeth from eight fly species. Regions where EDS measurements were made (1–3) are shown as R. Region 1 pertains to the location on the labellum between the pseudotracheae, and Region 2 and 3 are at the distal and proximal locations of the prestomal teeth, respectively.

Species	R	Na	S	Cl	K	Ca	Mg
*C. vomitoria*	1	0.15 ± 0.03Aa	1.31 ± 0.51a	0.20 ± 0.18B	0.99 ± 0.47Aa	0.12 ± 0.06a	0.05 ± 0.02ABCa
	2	0.07 ± 0.01Ab	0.08 ± 0.02BCb	0.04 ± 0.01B	0.07 ± 0.01Ab	0 ± 0Bb	0.01 ± 0.01b
	3	0.07 ± 0.02Ab	0.17 ± 0.04ABb	0.18 ± 0.04AB	0.16 ± 0.04ABb	0 ± 0Bb	0 ± 0b
*L. sericata*	1	0.05 ± 0.02C	1.27 ± 0.72	0.08 ± 0.07B	0.10 ± 0.03Ba	0.61 ± 0.53	0.02 ± 0.01C
	2	0.05 ± 0.03AB	0.08 ± 0.01BC	0.03 ± 0B	0 ± 0Bb	0 ± 0B	0 ± 0
	3	0.01 ± 0.01C	0.12 ± 0.02B	0.07 ± 0.01B	0.01 ± 0.01Bb	0 ± 0B	0 ± 0
*Pr. azurea*	1	0.06 ± 0.02BCa	0.42 ± 0.13a	0.01 ± 0.01Bb	0.13 ± 0.05B	0.06 ± 0.03a	0.05 ± 0.02Aa
	2	0.01 ± 0.01BCb	0.08 ± 0.01BCb	0.02 ± 0.01Bb	0.01 ± 0.0B	0 ± 0Bb	0 ± 0b
	3	0.03 ± 0.02BCab	0.15 ± 0.02ABb	0.16 ± 0.03ABa	0.04 ± 0.01B	0 ± 0Bb	0 ± 0b
*Sa. bullata*	1	0.12 ± 0.02ABa	0.58 ± 0.24	0 ± 0Bb	0.61 ± 0.29ABa	0.01 ± 0.01	0.07 ± 0.01A
	2	0.03 ± 0.01ABCb	0.09 ± 0.01BC	0.04 ± 0.01Bb	0.06 ± 0.01Ab	0 ± 0B	0 ± 0
	3	0.05 ± 0.01ABb	0.47 ± 0.33A	0.25 ± 0.12Aa	0.75 ± 0.61Ab	0 ± 0B	0 ± 0
*Po. vagabunda*	1	0 ± 0C	0.60 ± 0.36	0.01 ± 0.01B	0 ± 0B	0 ± 0	0.07 ± 0.01ABa
	2	0 ± 0C	0.13 ± 0.02B	0.02 ± 0.01B	0 ± 0B	0 ± 0B	0.01 ± 0.01b
	3	0 ± 0C	0.13 ± 0.04AB	0.10 ± 0.05AB	0 ± 0B	0 ± 0B	0 ± 0b
*M. domestica*	1	0.03 ± 0.02C	0.97 ± 0.51	0 ± 0Ba	0.25 ± 0.13B	0.40 ± 0.28	0.03 ± 0.02ABC
	2	0 ± 0C	0.22 ± 0.04A	0.12 ± 0.03Aab	0.06 ± 0.02A	0.05 ± 0.03A	0 ± 0
	3	0.01 ± 0.01	0.27 ± 0.08AB	0.20 ± 0.07ABb	0.04 ± 0.02B	0.03 ± 0.02A	0.01 ± 0.01
*St. calcitrans*	1	0 ± 0C	0.97 ± 0.31a	1.66 ± 1.04A	0.06 ± 0.06B	0.05 ± 0.05	0 ± 0C
	2	0 ± 0C	0.10 ± 0.06BCb	0.04 ± 0.02B	0 ± 0B	0 ± 0B	0 ± 0
	3	0 ± 0C	0.10 ± 0.03ABb	0.20 ± 0.09AB	0 ± 0B	0 ± 0B	0 ± 0
*Sc. stercoraria*	1	0.01 ± 0.01C	0.35 ± 0.13a	0 ± 0Bb	0 ± 0B	0.02 ± 0.01	0.02 ± 0.01BC
	2	0 ± 0C	0.04 ± 0.01Cb	0 ± 0Bb	0 ± 0B	0 ± 0B	0 ± 0
	3	0 ± 0C	0.10 ± 0.03ABb	0.20 ± 0.03ABa	0 ± 0B	0 ± 0B	0 ± 0

Uppercase letters represent significant differences among species per region, lowercase letters represent differences among regions within species.

**Table 4:** Linear discriminant analysis classification of fly species for each studied region. The numbers correspond to Regions 1, 2, and 3, respectively. The results for Region 2 (distal prestomal teeth measurements) are bolded to indicate their relatively accurate classification results.

Species	C. v.	L. s.	M. d.	P. v.	P. a.	S. b.	S. s.	S. c.
C. v.	4, ***4***, 5	0, 0, 0	0, 0, 0	0, 0, 1	0, 0, 0	1, 1, 0	0, 1, 0	0, 0, 0
L. s.	0, 0, 1	3, ***3***, 3	0, 0, 0	0, 0, 2	1, 1, 0	1, 0, 0	1, 1, 0	0, 1, 0
M. d.	0, 0, 0	0, 0, 2	1, ***5***, 2	1, 0, 2	1, 0, 0	1, 1, 0	2, 0, 0	0, 0, 0
P. v.	0, 0, 0	0, 0, 1	0, 0, 0	3, ***2***, 2	0, 1, 0	0, 0, 0	0, 0, 0	0, 0, 0
P. a.	0, 0, 2	0, 0, 1	0, 0, 0	1, 0, 1	2, ***5***, 1	1, 0, 0	2, 1, 0	0, 0, 1
S. b.	1, 1, 2	0, 0, 0	0, 0, 0	0, 0, 0	1, 0, 0	3, ***4***, 3	0, 0, 0	0, 0, 0
S. s.	0, 0, 0	0, 0, 0	0, 0, 0	0, 0, 0	1, 0, 0	0, 0, 0	3, ***4***, 3	0, 0, 1
S. c.	0, 0, 0	0, 0, 1	0, 0, 0	0, 0, 0	0, 1, 0	0, 0, 0	2, 0, 2	1, ***2***, 0

C. v. = *Calliphora vomitoria*, L. s. = *Lucilia sericata*, M. d. = *Musca domestica*, P. v. = *Pollenia vagabunda*, P. a. = *Protocalliphora azurea*, S. b. = *Sarcophaga bullata*, S. s. = *Scathophaga stercoraria*, S. c. = *Stomoxys calcitrans*.

## Data Availability

The data presented in this study are available on request from the corresponding author.

## Supplementary Materials

The following supporting information can be downloaded at: https://www.mdpi.com/article/10.3390/insects13020207/s1, Table S1. ANOVA results of comparisons of the elemental analysis among regions within each species. Table S2. ANOVA results of comparisons of the elemental analysis within regions among each species.
