# Supplementary material for: Material Properties and Morphology of Prestomal Teeth in Relation to the Feeding Habits of Diptera (Brachycera)
Source: Insects. 2022 Feb 17;13(2):207. doi: 10.3390/insects13020207 (PMC8880167; doi:10.3390/insects13020207)
Supplement: Supplementary file 1 [file insects-13-00207-s001.zip › insects-1586831-supplementary.pdf]

Supplementary Table S1. ANOVA results of comparisons of the elemental analysis among regions within each species.

Species	Element	df	F	p value
C. vomitoria	Na	2, 15	4.0922	0.0382*
	S	2, 15	5.3222	0.0179*
	Ca	2, 15	3.9706	0.0413*
	Mg	2, 15	3.9796	0.0411*
	P	2, 15	5.675	0.0146*
	Cl	2, 14	0.8766	0.4378
	K	2, 15	3.4252	0.0595*
L. sericata	Na	2, 15	1.1552	0.3415
	S	2, 15	2.6557	0.1029
	Ca	2, 15	1.334	0.2929
	Mg	2, 15	2.4545	0.1196
	P	2, 15	12.6332	0.0006*
	Cl	2, 15	0.6048	0.559
	K	2, 15	7.906	0.0045*
Pr. azurea	Na	2, 15	2.2829	0.1363
	S	2, 15	5.8307	0.0134*
	Ca	2, 15	4.0935	0.0381*
	Mg	2, 15	9.8941	0.0018*
	P	2, 15	9.54	0.0021*
	Cl	2, 15	13.7965	0.0004*
	K	2, 15	5.2153	0.0191*
Sa. bullata	Na	2, 12	12.2766	0.0013*
	S	2, 12	1.1649	0.3449
	Ca	2, 12	1	0.3966
	Mg	2, 12	27.9277	< 0.0001*
	P	2, 12	0.9795	0.4036
	Cl	2, 12	3.9845	0.0471*
	K	2, 12	0.8756	0.4416
Po. vagabunda	Na	2, 6	NA	NA
	S	2, 6	1.6571	0.2673
	Ca	2, 6	NA	NA
	Mg	2, 6	33.0909	0.0006*
	P	2, 6	1.6946	0.261
	Cl	2, 6	2.5936	0.1543
	K	2, 6	NA	NA
M. domestica	Na	2, 15	1.1446	0.3446
	S	2, 15	2.0108	0.1684
	Ca	2, 15	1.6701	0.2214
	Mg	2, 15	2.325	0.132
	P	2, 15	1.8997	0.1839
	Cl	2, 15	4.4581	0.0302*
	K	2, 15	2.3633	0.1282
Sc. stercoraria	Na	2, 9	1	0.4053
	S	2, 9	4.5099	0.044*
	Ca	2, 9	2	0.1911
	Mg	2, 9	2.6667	0.1232
	P	2, 9	9	0.0071*
	Cl	2, 9	28.6477	0.0001*
	K	2, 9	NA	NA
St. calcitrans	Na	2, 6	NA	NA

S	2, 6	7.4248	0.0238*
Ca	2, 6	0.9336	0.4436
Mg	2, 6	NA	NA
P	2, 6	1	0.4219
Cl	2, 6	2.2115	0.1908
K	2, 6	1	0.4219

* indicates significant differences (< 0.05), NA = not applicable and occurs when there were no values recorded

Supplementary Table S2. ANOVA results of comparisons of the elemental analysis within region among each species.

Region	Element	df	F	p value
1	Na	7, 31	5.6465	0.0003
	Cl	7, 30	4.393	0.0019
	S	7, 31	0.6569	0.706
	K	7, 31	2.2719	0.0546
	Ca	7, 31	0.7745	0.6131
	Mg	7, 31	2.0912	0.0745
	P	7, 31	1.2465	0.3084
2	Na	7, 31	2.5132	0.0361
	Cl	7, 31	4.1934	0.0024
	S	7, 31	5.35	0.0004
	K	7, 31	9.5077	< 0.0001
	Ca	7, 31	2.352	0.0476
	Mg	7, 31	0.955	0.4802
	P	7, 31	3.4901	0.0007
3	Na	7, 31	4.5711	0.0013
	Cl	7, 31	0.8772	0.5354
	S	7, 31	0.9773	0.465
	K	7, 31	1.3467	0.2625
	Ca	7, 31	1.3626	0.2558
	Mg	7, 31	0.6837	0.6847
	P	7, 31	1.3787	0.2491

* indicates significant differences (< 0.05), NA = not applicable and occurs when there were no values recorded
